# Parent-teacher agreement on ADHD symptom ratings: a systematic review and meta-analysis

**DOI:** 10.3389/fpsyt.2026.1770305

**Published:** 2026-09-28

**Authors:** Qi-Qi Fu, Dan-Hui Chen, Ze-Ping Shen, Qian Zhuang, Hang Zhang, Matthew Francis Lock, Na Zhao, Yu-Feng Zang

**Affiliations:** 1 Centre for Cognition and Brain Disorders/Department of Neurology, The Affiliated Hospital of Hangzhou Normal University, Hangzhou, Zhejiang, China; 2 Precision Transcranial Magnetic Stimulation (TMS) Center, The Affiliated Hospital of Hangzhou Normal University, Hangzhou, Zhejiang, China; 3 Institutes of Psychological Sciences, Hangzhou Normal University, Hangzhou, Zhejiang, China; 4 Zhejiang Key Laboratory for Research in Assessment of Cognitive Impairments, Hangzhou Normal University, Hangzhou, China; 5 Center for Cognition and Brain Disorders, Hangzhou Normal University, Hangzhou, China; 6 Center for Psychological Sciences, Zhejiang University, Hangzhou, Zhejiang, China; 7 Engineering Research Center of Mobile Health Management System, Ministry of Education, Hangzhou Normal University, Hangzhou, China

**Keywords:** attention-deficit/hyperactivity disorder, kappa statistic, meta−analysis, parent–teacher agreement, symptom rating scale

## Abstract

**Background/objectives:**

Accurate diagnosis of attention-deficit/hyperactivity disorder (ADHD) requires multi-informant assessments across different settings, yet consistent agreement between parents and teachers remains challenging. This systematic review and meta-analysis aimed to quantify the level of concordance between parent and teacher ratings of ADHD symptoms in children and adolescents and to examine the moderating effects of child gender and sample settings.

**Methods:**

We conducted a comprehensive search of Web of Science, Embase, and PubMed from 1994 to May 2026(PROSPERO: CRD42024556281). Inclusion criteria encompassed studies assessing ADHD symptoms in individuals under 18 years using DSM-IV/V criteria and reporting Cohen’s Kappa for parent-teacher agreement. A random-effects meta-analysis was performed, with subgroup analyses based on gender and sample settings (clinical, school, community). Heterogeneity was assessed using the I² statistic.

**Results:**

Eleven studies involving 8796 participants were included. The overall parent-teacher agreement was low (Kappa = 0.25, 95% CI: 0.18-0.31), with substantial heterogeneity (I² = 75.8%). Subgroup analyses revealed that gender had a minimal impact on agreement levels (boys: Kappa=0.25; girls: Kappa=0.32). However, sample settings significantly moderated agreement: school-based samples showed the highest concordance (Kappa=0.31), followed by community samples (Kappa=0.27), while clinical samples demonstrated the lowest and non-significant agreement (Kappa=0.07).

**Conclusions:**

This meta-analysis confirmed low parent-teacher agreement in ADHD assessments, with the lowest agreement observed in clinical samples. Existing studies also suggest moderate agreement between parents themselves, although the limited data precluded a meta-analysis on this specific comparison. Nevertheless, these findings highlight that high levels of discrepancy across informants should be a focus of clinical attention. When integrating multi-informant data, such discrepancies must be explicitly interpreted, and standardized methods are needed to reconcile them in diagnostic decision-making.

**Systematic review registration:**

https://www.crd.york.ac.uk/prospero/display_record.php?RecordID=4556281, identifier CRD4202455628.

## 1 Introduction

Attention-deficit/hyperactivity disorder(ADHD) is a developmental disorder marked by enduring and widespread symptoms of inattention, hyperactivity, and impulsivity that impacts an individual’s performance in daily life (1). Diagnosis necessitates the manifestation of symptoms in a minimum of two separate contexts. The initial phase in the diagnostic procedure is completing a rating scale to get input (2) from several informants, often parents and teachers (3). Achieving sufficient interrater reliability across informants has always proved difficult due to poor correlations and discrepancies among parent and teacher evaluations, thus there are concerns regarding the reliability and validity of ADHD scales (4–6).

Parents and teachers, due to their differing perspectives and roles, often form distinct standards and expectations when assessing a child’s behavior. Parental reports of child behavior tend to reflect subjective, informant-specific perspectives. On the other hand, teachers simultaneously engage with several children of similar age and differing backgrounds, giving them a unique perspective to recognize abnormal behaviors in a specific age group (6). Consequently, parents and teachers may establish disparate standards, not only due to their observations of children’s conduct in varied contexts, but also owing to their distinct reasons for assigning ratings or differing expectations regarding normative and atypical behavior (7, 8).

The reliability of ADHD diagnosis through rating scales is fundamentally challenged by inconsistencies among informant reports. However, the evaluations performed by clinicians and researchers differ based on the selected informant, the level of concordance across informants, and their priority ranking when information conflicts occur (9). The examination of an individual informant’s rating validity is complicated because no established approach to assessing the severity of ADHD symptoms in children exists (4). Moreover, variations across informants indicate that a single informant or an amalgamation of data from several informants may result in different findings on the significance or presence of ADHD (10). Consequently, inconsistencies in informant accounts may lead to inconsistent outcomes in the diagnosis, prevalence, and related comorbidities of ADHD.

Identifying the key factors affecting informant agreement is critical for improving ADHD diagnostic accuracy. This would aid in delivering evidence-based techniques so researchers and practitioners can integrate and interpret feedback from various informants during ADHD screening (11). Previous research has examined many factors influencing the consistency across informants, including child attributes such as age, gender, and ethnicity (12, 13), as well as informant-related attributes including education level and psychological condition (14). Gender has also been recognized as a possible factor, with ADHD allegedly more prevalent among boys. Likewise, informant attributes, including parental education and mental health, have been shown to influence the reliability of ADHD assessments. Furthermore, it is necessary to investigate whether the context from which samples are obtained affects the degree of concordance among informants.

This systematic review and meta-analysis seek to evaluate three primary hypotheses: (1) there is a low concordance among father, mother, and teacher assessments of ADHD, (2) child gender is associated with the degree of concordance between these assessments, and (3) different sample settings (community, clinical, or school-based) are associated with varying levels of concordance between informants.

## 2 Materials and methods

This systematic review and meta-analysis was conducted in accordance with the guidelines of the Preferred Reporting Items for Systematic Reviews and Meta-analyses (PRISMA) statement (see **Supplementary Tables S1**, **S2**). The review protocol was prospectively registered with PROSPERO (registration number: CRD4202455628).

### 2.1 Search methodology

We conducted a search across Embase, Web of Science, and PubMed to incorporate papers published from 1994 to May 2026. The search was initially completed on August 28, 2025, and was subsequently updated on May 31, 2026, using an identical search strategy. No additional studies meeting the predefined inclusion criteria were identified in the updated search.We devised the PubMed search technique and subsequently modified it for additional databases: ((((attention deficit disorder with hyperactivity[MeSH Terms]) OR (ADHD[MeSH Terms])) OR (attention deficit hyperactivity disorder[MeSH Terms])) AND ((((assessment) OR (assess)) OR (diagnosis)) AND (rating scales)) AND (parent) AND (teacher))) AND ((((agreement)) OR (consistency)) OR (coherence)). The Endnote program was utilized to consolidate the reference list and eliminate duplicate entries.

### 2.2 Criteria for selection

This meta-analysis was comprised of cross-sectional studies that provided data about the concordance between parents and teachers regarding ADHD symptom assessments.

The criteria for inclusion are as follows:

1. Population: individuals under 18 years of age from various countries and ethnic/racial backgrounds;
2. Study design: Cross-sectional and observational studies;
3. Comparators: Diagnoses of ADHD were confirmed by qualified mental health professionals (e.g., psychiatrists or clinical psychologists) based on DSM criteria. The diagnostic process typically incorporated established rating scales or semi-structured interviews;
4. Comorbidity: Studies including children with comorbid conditions or ADHD subtypes were included to reflect real-world clinical practice;
5. Presents correlational data (e.g. Cohen’s kappa) to evaluate our correlational hypothesis;
6. Published in English;
7. Published from 1994 until May 2026.

The Diagnostic and Statistical Manual of Mental Disorders (DSM-IV; 4th edition), released in 1994, established 18 diagnostic criteria for ADHD. To guarantee that all included research conformed to either the DSM-IV or DSM-V diagnostic criteria, the commencement year for this study was established as 1994.

### 2.3 Screening

Endnote 21 was employed to arrange references, eliminate duplicate entries, and monitor screening choices. Following the removal of duplicate articles, an initial screening of titles and abstracts was conducted to exclude publications that failed to match the inclusion criteria. A second screening phase was conducted by reviewing the whole content of the articles. **Figure 1** is a flow diagram charting the process followed for retrieving the relevant works. Throughout both screening phases, prospective papers for inclusion were assessed separately by two reviewers. A third reviewer served as the arbitrator who settled disputes through discussions.

**Figure 1:**
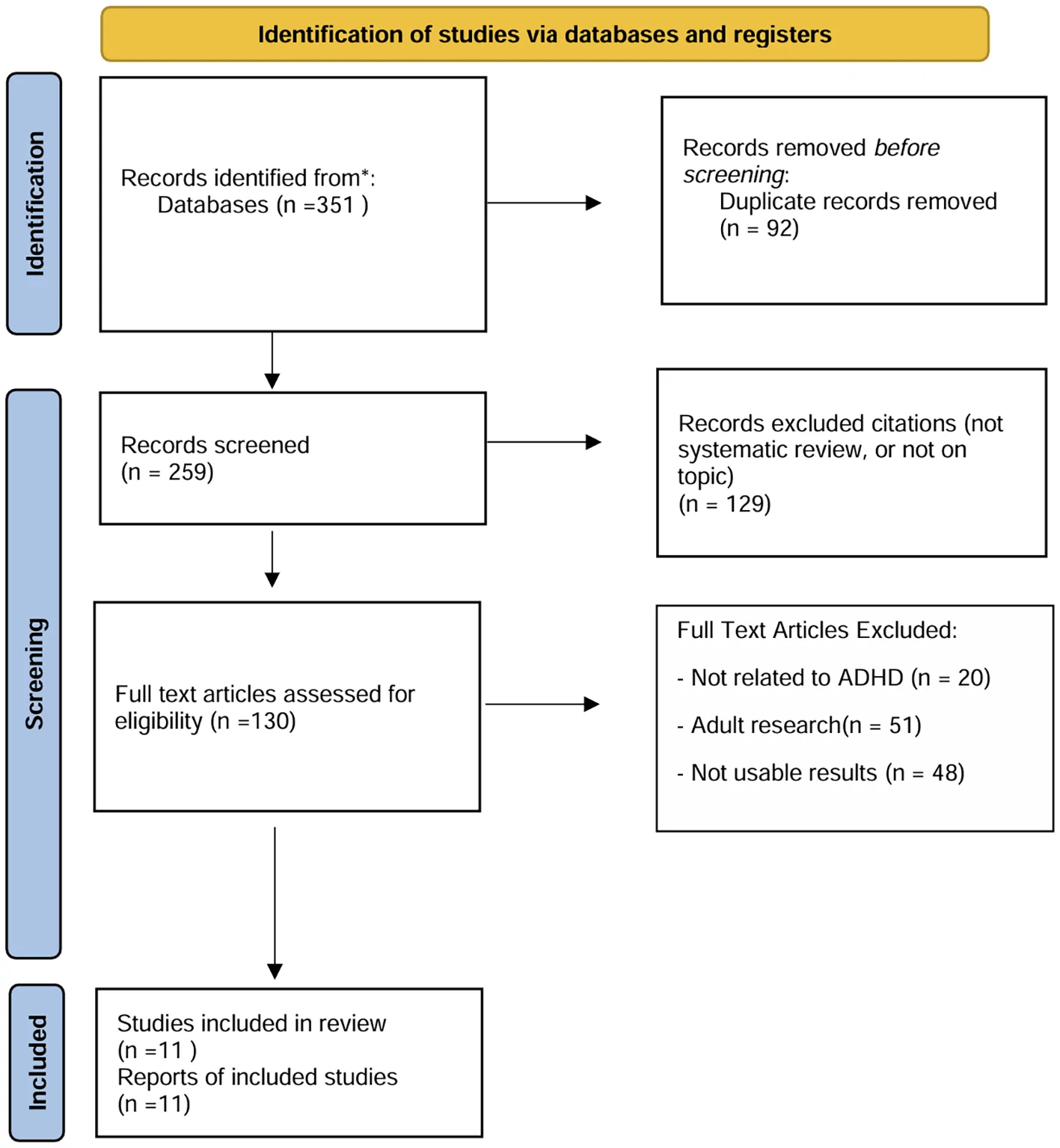
PRISMA flow chart depicting the articles obtained from the first search and the subsequent article screening procedure.

### 2.4 Extraction of data and evaluation of quality

Included papers were evaluated for pertinent information included in the meta-analysis, together with the constraints of their findings.In the data extraction phase, information was gathered for generic factors (e.g., authors, publication year); sample specifics (e.g., sample size, mean, age); methodology (e.g., assessment instruments); and statistical metrics (e.g., effect sizes). In the meta-analytic technique, we treated the effect sizes of subgroups (e.g., gender, sample settings) as independent samples. All rating scales employed to evaluate the severity of ADHD in the selected studies were developed and validated by previous research.The subsequent data was extracted into an Excel spreadsheet by two independent reviewers (see **Table 1**).

**Table 1:** Main information of studies.

Author	Sample size	Sample settings	Assessment tools	Boy/girl	Mean age	Cohen’s kappa
Hei et al. (2024) (11)	7977	community	SDQ	4111/3866	10.54	k= 0.34
Kristin et al. (2023) (15)	328	community	ADHD-RS	—	8	k= 0.22
Akın et al. (2021) (16)	417	school	VADRS	225/192	9.18	k=0.367
Yaira et al. (2021) (17)	789	school	ADHD-RS	454/335	8.4	k=0.3
Margaret et al. (2017) (18)	70	school	VADRS/IRS	47/23	8.13	k=0.33
Debbie et al. (2017) (19)	200	school	SDQ	98/102	4.62-4.84	k=0.23
Alan et al. (2017) (20)	899	community	CBCL/TRF	428/471	10	k=0.23
Kumi et al. (2016) (21)	69	clinical	ADHD-RS	50/19	9.5	k=-0.09
Prahbhjot et al. (2008) (22)	119	clinical	CPRS/CTRS	103/16	8.4	k= 0.08
Pieter et al. (2004) (6)	30	clinical	CBCL/TRF	24/6	10.4	k=0.10
Effie et al. (2000) (2)	74	clinical	DBD	60/14	8.9	k=0.2

SDQ, Strengths and Difficulties Questionnaire; ADHD-RS, Attention-Deficit/Hyperactivity Disorder Rating Scale; VADRS, Vanderbilt ADHD Diagnostic Rating Scale; IRS, Impairment Rating Scale; CBCL, Child Behavior Checklist; TRF, Teacher Report Form; CPRS, Comprehensive Parent Rating Scale; CTRS, Conners’ Teacher Rating Scale; DBD, Disruptive Behavior Disorder Rating Scale. The en dash (—) indicates that the corresponding data were not reported in the original study.

### 2.5 Risk of bias analysis

This systematic review and meta-analysis assessed the quality of the included observational studies with a modified QUADAS-2 technique, which is specifically intended to evaluate the risk of bias in diagnostic accuracy studies. We conducted an independent assessment of each study based on the four fundamental components of QUADAS-2: patient selection, index test, reference standard, and flow and timing, in addition to applicability concerns. Comprehensive quality assessments were conducted, and each domain was rated as having a ‘low risk’, ‘high risk’, or ‘unclear risk’ of bias (see **Supplementary Table S3**). A detailed list of the modified QUADAS-2 signaling questions used in this systematic review is available in the **Supplementary Material** (**Supplementary Table S4**).

### 2.6 Statistical evaluation

All papers presenting pertinent findings chose Cohen’s Kappa coefficient as the aggregated effect size for the meta-analysis. The analysis of the study was conducted using Stata 16.0 software. A meta-analysis was conducted employing a random-effects model, and the aggregated effect sizes together with their 95% confidence intervals were computed. The I² statistic was utilized to evaluate heterogeneity, with thresholds of 25%, 50%, and 75% indicating low, moderate, and high heterogeneity, respectively. We performed a statistical sensitivity analysis (using a leave-one-out exclusion procedure) to evaluate the robustness of the meta-analysis findings. This process helps determine the influence of individual studies on the overall pooled effect size, ensuring the results are not driven by a single outlier. This systematic study used subgroup analyses to examine the impact of various factors on the alignment of parent and teacher evaluations of ADHD. Subgroups were delineated based on participant gender and sample settings (clinical/school/community). Forest plots were employed to illustrate effect sizes across studies along with 95% confidence intervals. Publication bias was assessed by funnel plots and Egger’s test (**Supplementary Figure S1** in the **Supplementary Material**).

## 3 Results

### 3.1 Characteristics of the study

The search produced 351 potential articles, of which eleven satisfied our inclusion criteria. Sample sizes varied from 30 to 7,977 people. The mean age of participants across the eleven studies ranged from 4.62 to 10.54 years. Ten studies included the gender ratio, with the proportion of boys varying from 47% to 87%. Of the eleven investigations, four’s utilized clinical samples, four concentrated on school-based samples, and three included community samples. Correlation forest plots were employed to depict the impacts of each study across various dimensions (**Figures 2**–**5**).

**Figure 2:**
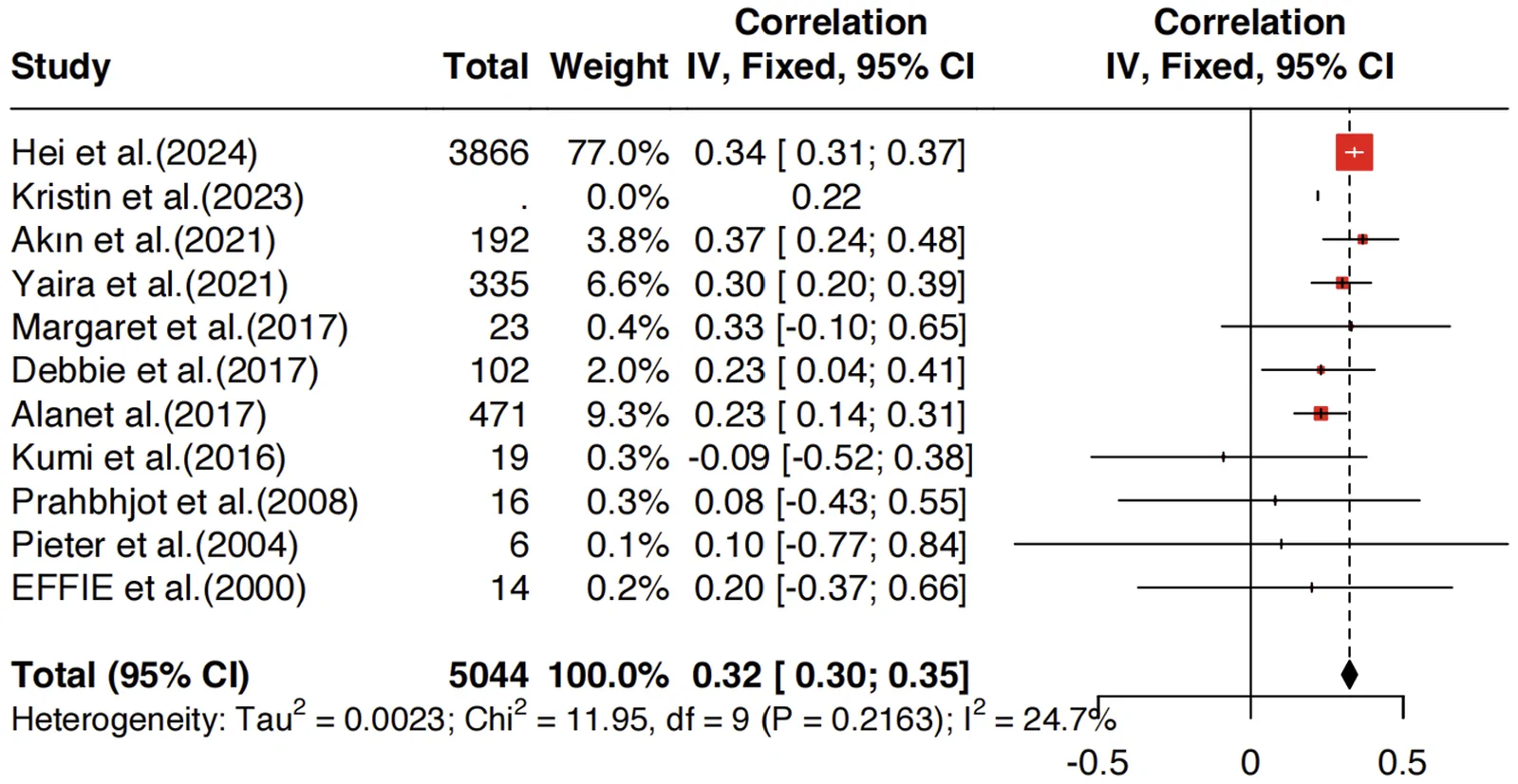
Forest plot depicting parent-teacher concordance on ADHD symptom evaluation.

**Figure 3:**
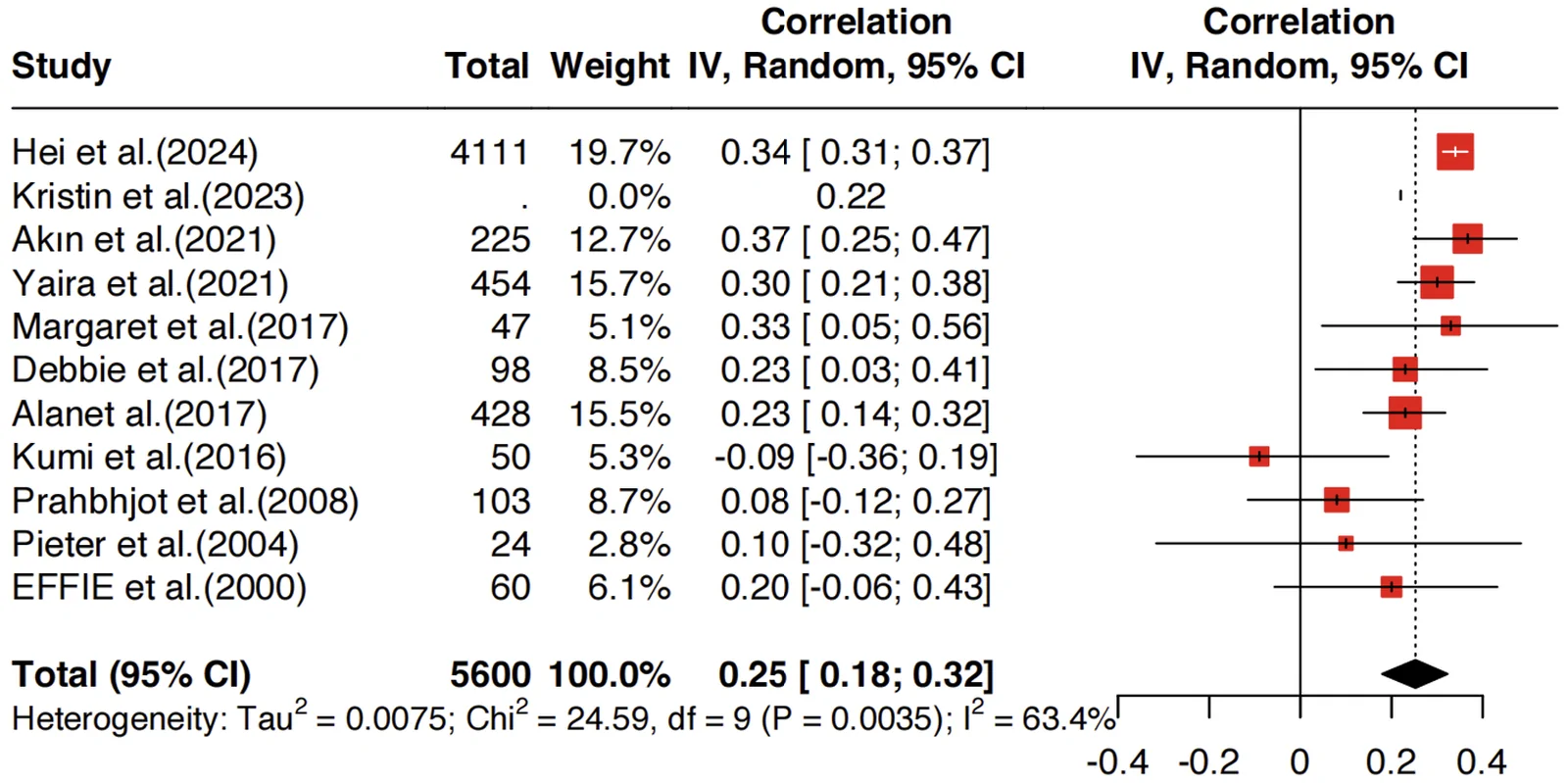
Forest plot for subgroup analysis: boys.

**Figure 4:**
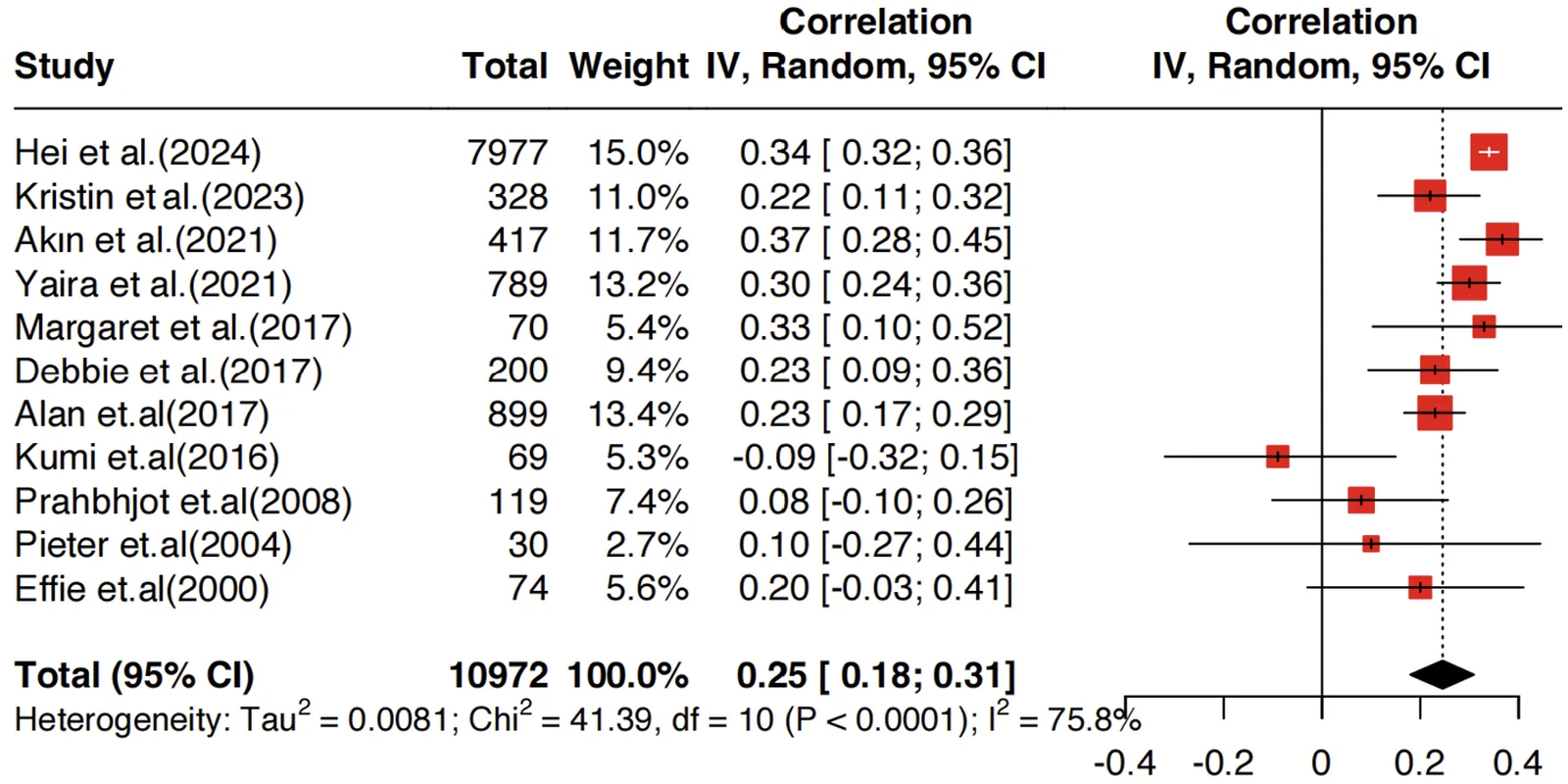
Forest plot of subgroup analysis: girls.

**Figure 5:**
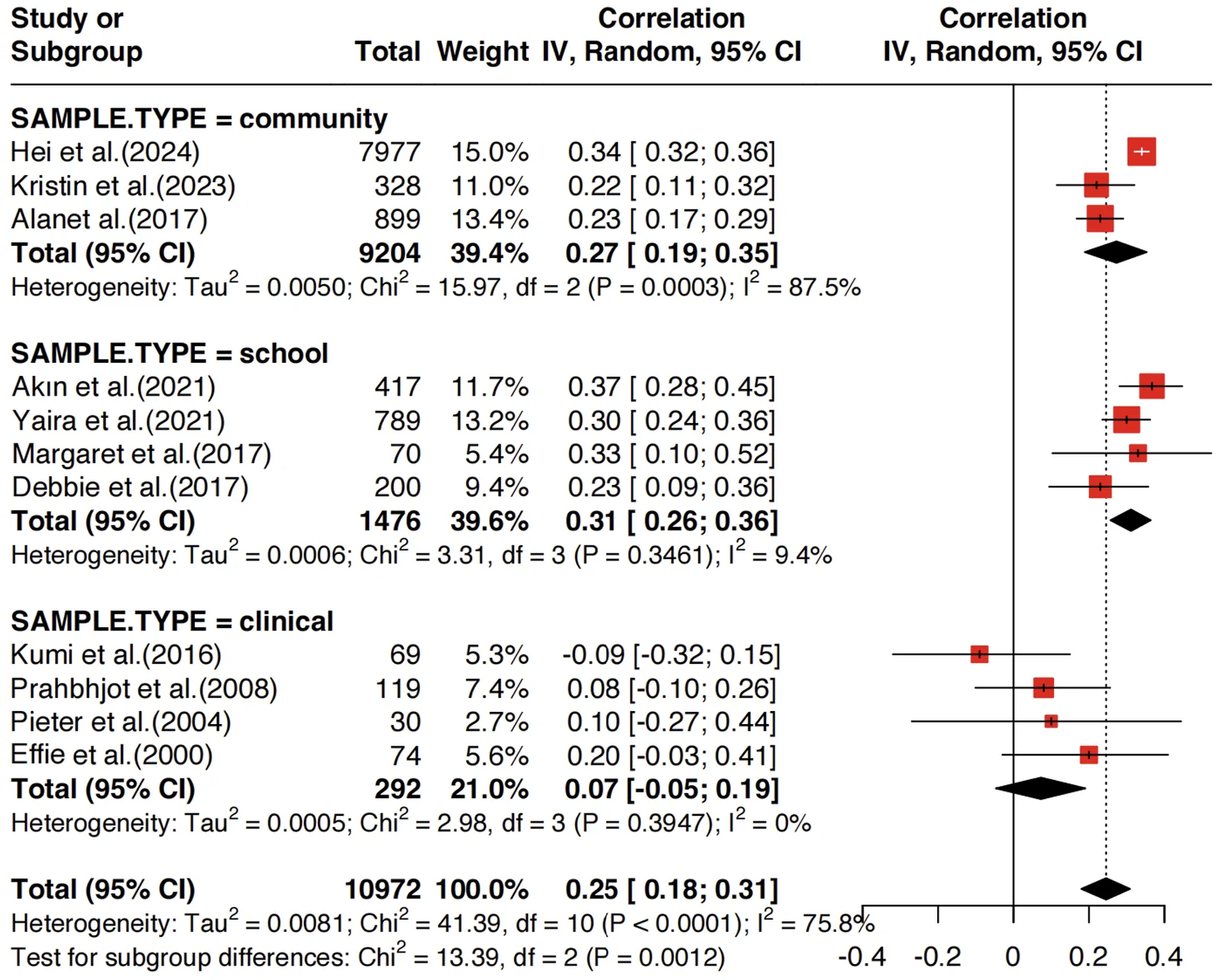
Forest plot of subgroup analysis --sample settings (parent–teacher agreement on ADHD symptoms: highest in school, intermediate in community, lowest (non-significant) in clinical samples).

All studies utilized DSM-IV (23), DSM-IV(TR) (24), or DSM-V (25) ADHD diagnostic criteria and implemented structured or semi-structured clinical interviews as the definitive diagnostic evaluation. Assessment instruments employed to gather data from parents and teachers for the purpose of predicting the gold standard diagnosis varied across studies (detailed in **Table 1**). These instruments included the Vanderbilt ADHD Diagnostic Rating Scale (VADRS), ADHD Rating Scale (ADHD-RS), Impairment Rating Scale (IRS), Child Behavior Checklist (CBCL), Comprehensive Parent Rating Scale (CPRS), Teacher Report Form (TRF), Connor’s Teacher Rating Scale (CTRS), Disruptive Behavior Disorder Rating Scale (DBD), and Strengths and Difficulties Questionnaire (SDQ).

### 3.2 Concordance between parent and teacher evaluations of ADHD symptoms

Quantitative assessments of the principal outcomes were performed, encompassing 11 trials with a cumulative total of 8796 individuals. The aggregated effect size was k = 0.25 (95% CI: 0.18-0.31), p < 0.0001. This indicates a poor concordance between parents’ and teachers’ evaluations of ADHD symptoms. The study revealed a heterogeneity test result of I² = 75.8%, indicating substantial heterogeneity. The forest plot (**Figure 2**) presents the comprehensive results. A leave-one-out sensitivity analysis confirmed the robustness of our findings, demonstrating that the exclusion of any single study did not result in a statistically significant shift in the overall pooled effect size. To address the significant heterogeneity shown in the meta-analysis results, we performed subgroup analyses categorized by gender and sample settings in the subsequent phase to elucidate the variables contributing to the variability among studies.

### 3.3 Impact of gender on the concordance of ADHD evaluations between parents and teachers

Subgroup analyses of gender were conducted utilizing the 10 studies that supplied comprehensive counts of boys and girls. The comprehensive findings are presented in the forest plot, with **Figure 3** illustrating the outcomes for boys and **Figure 4** for girls. The Kappa coefficient for the group of girls, including a total sample size of 5044, was 0.32 (95% CI: 0.30-0.35), signifying modest heterogeneity (I² = 24.7%). Conversely, for the boys’ cohort, comprising 5600 participants, the Kappa coefficient was 0.25 (95% CI: 0.18-0.32), exhibiting significant heterogeneity (I² = 63.4%). The results indicate that gender may influence the consistency of ADHD evaluation, but is perhaps not a determining factor.

### 3.4 Impact of sample settings on parent and teacher concordance in ADHD evaluation

The subgroup analyses showed that varying sample environments significantly influenced the correlation (p < 0.0001) (**Figure 5**): the highest concordance was noted in the school sample (k = 0.31, 95% CI: 0.26-0.36), succeeded by the community sample (k = 0.27, 95% CI: 0.19-0.35). The clinical sample had the smallest connection, which was not statistically significant (k = 0.07, 95% CI: -0.05-0.19). The research results revealed that variations in sample settings significantly influence the level of concordance, thus it seems important to include and analyzing of sample features and their contexts in informant agreement investigations to ensure a reliable outcome.

### 3.5 Sensitivity analysis and publication bias

Leave-one-out sensitivity analysis. To assess the robustness of the pooled estimates, we performed leave-one-out sensitivity analyses for the overall analysis and for each gender subgroup. In the overall analysis, the pooled kappa remained statistically significant (all p < 0.0001) and directionally consistent across all omission scenarios, confirming that no single study exerted undue influence on the overall findings.

For the boys’ subgroup, the fixed-effects pooled estimate remained stable at approximately 0.32 [0.29–0.34] across all leave-one-out scenarios, with the exception of omitting Hei Ka Chan, which yielded a slightly lower estimate of 0.25 [0.21–0.30]. The I² statistic ranged from 46.5% to 67.5% across scenarios, indicating that moderate-to-high heterogeneity persisted in the boys’ subgroup regardless of individual study removal.

For the girls’ subgroup, the fixed-effects pooled estimate was highly consistent at approximately 0.32 [0.30–0.35] in all scenarios (all p < 0.0001), with I² values ranging from 0% to 33.0%, confirming the robustness and relative homogeneity of the girls’ subgroup estimate.

Publication bias. Publication bias was assessed using funnel plot inspection and Egger’s linear regression test. Egger’s test indicated statistically significant funnel plot asymmetry in the overall sample (t = −3.34, df = 9, p = 0.009; bias estimate = −2.00, SE = 0.60), the boys’ subgroup (t = −3.05, df = 8, p = 0.016; bias estimate = −1.62, SE = 0.53), and the girls’ subgroup (t = −2.34, df = 8, p = 0.048; bias estimate = −0.90, SE = 0.39). These negative bias estimates suggest that smaller studies reporting lower kappa values may be underrepresented in the literature, potentially indicating some degree of publication bias.

However, these findings must be interpreted with caution. With only 11 studies included, Egger’s test has limited statistical power and may produce unreliable results, as formal tests of funnel plot asymmetry generally require at least 10–15 studies to yield valid inferences. Furthermore, funnel plot asymmetry may reflect sources other than publication bias, including true between-study heterogeneity, small-study effects, or population-level differences. Nonetheless, the possibility of publication bias cannot be completely excluded, and the magnitude of the pooled estimates should be interpreted accordingly.

## 4 Discussion

This systematic review included 11 eligible studies. The random-effects meta-analysis showed low overall concordance between parent and teacher ratings of ADHD symptoms in children and adolescents (pooled Cohen’s κ = 0.25; 95% CI: 0.18–0.31), with substantial between-study heterogeneity (I² =75.8%). Subgroup analyses indicated that gender had a limited effect on cross-informant agreement (girls: κ = 0.32; boys: κ = 0.25), whereas sample settings was a significant moderator: school-based samples exhibited the highest agreement (κ = 0.31), community samples were intermediate (κ = 0.27), and clinical samples showed the lowest, non-statistically significant agreement (κ = 0.07).

Overall, the evidence supports low-to-moderate cross-context concordance between parent and teacher reports. This aligns with our hypothesis and prior evidence indicating that cross-informant agreement is often low to moderate (26). This finding carries direct theoretical and clinical implications for multi-informant ADHD diagnosis and for strategies to integrate discrepant reports.

The secondary objective of our study was to examine if variations in children’s gender and sample settings would result in increased or decreased cross-informant concordance. Our findings revealed a modest numerical difference between gender subgroups (girls: κ = 0.32; boys: κ = 0.25); however, this difference should be interpreted descriptively, as no formal test of subgroup differences was conducted for gender. The available evidence is therefore insufficient to support gender as a robust moderator of parent–teacher agreement. This aligns with prior research by Briggs (1996) and Kolko (1993), which similarly found no systematic gender influence on cross-informant congruence at the scale level. Notably, the heterogeneity in the boys’ subgroup (I² = 63.4%) was considerably higher than in the girls’ subgroup (I² = 24.7%), suggesting that agreement levels may be more variable across contexts for boys. This may partly reflect the higher prevalence and more variable symptom phenotype of ADHD in boys, though this hypothesis warrants further investigation in future studies with larger, more balanced gender samples. This aligns with research by Briggs (27) and Kolko (28), which similarly found no gender influence on opinion congruence between parents and teachers at the scale level.

The findings of this meta-analysis on the influence of sample settings on parent-teacher assessment concordance aligned with our hypotheses. The concordance between parent and teacher assessments was markedly poorer in the clinical samples than in the school and community samples. The current study specifically suggests that informant agreement was least robust in the clinical group. This outcome contradicts prior comparable research, which generally posits increased cross-informant agreement in clinical samples due to the heightened severity and more apparent characteristics of ADHD symptoms in these groups (29). However, it is crucial to interpret these results cautiously due to the small sample sizes included in each of the studies. Several factors may account for this unexpected finding. First, clinical samples are typically more diagnostically heterogeneous, often including children with comorbid psychiatric conditions such as oppositional defiant disorder, anxiety disorders, autism spectrum disorder, or learning disabilities. These comorbidities may produce context-specific symptom expression that manifests differently at home versus school, thereby reducing cross-informant agreement. Second, referral pathways may introduce systematic sampling bias: children are frequently referred to clinical services because symptoms are prominently reported by one informant source—either parents or teachers—rather than because symptoms are consistently evident in both settings. Third, the four clinical studies included in this review used different assessment instruments (ADHD-RS, CPRS/CTRS, CBCL/TRF, and DBD), which differ in item content, rating formats, and diagnostic thresholds. These instrument-level differences may have further diminished apparent agreement independently of true informant discrepancy. Taken together, the low kappa in clinical samples should not be interpreted as contradicting clinical expectations of greater symptom severity in this population, but rather as evidence that severity does not necessarily translate into cross-informant consistency—especially when referral processes, comorbidity patterns, and measurement tools introduce systematic sources of variance.

In addition to the previously mentioned gender characteristics, child’s age, parental gender, age, educational attainment, socioeconomic situation, and the degree of parental depression also possible influence on the results. In the research examined, Hei ka Chan (11) determined that younger parents were more likely to report significant discrepancies with instructors, especially regarding evaluations of hyperactivity and inattention. Moreover, parents possessing better educational attainment or experiencing more severe sadness were more inclined to report increased ADHD symptoms in comparison to instructors. The depression distortion theory posits that parental depression adversely impacts the accuracy of parental assessment (30). Beyond depression, informant bias plays a significant role in discrepancies. Parents’ daily stress levels, differing parenting styles, and varying thresholds for tolerating disruptive behavior often subjectively tint their reports. Conversely, teachers evaluate children in structured academic environments and might be influenced by high classroom workloads, leading them to be more sensitive to behaviors that disrupt classroom management, such as hyperactivity, rather than subtle inattention. Furthermore, the rating scales themselves present inherent limitations, including vague wording of items, lack of situational context, and susceptibility to rater fatigue or recall bias. These findings further highlight the need for more holistic analyses of ADHD evaluations and why there are such large discrepancies between informants.

Consistent with the low parent-teacher agreement found in this meta-analysis, existing literature also reports significant differences between father and mother ratings of ADHD symptoms. For example, Sollie (31) demonstrated that mothers report higher levels of inattention symptoms, whereas Gomez (32) found that fathers tend to assign higher severity ratings for hyperactivity–impulsivity. Further corroborating this pattern, Caye (33) reported moderate interparental agreement overall, observing that mothers generally endorsed more symptoms than fathers and noting that differences in parental education levels further diminished agreement. However, owing to the limited number of studies that specifically quantify interparental concordance, a meta-analysis on this topic was not feasible in the present review. Several critical questions therefore remain to be addressed in future research—for instance, whether a report from only one parent is sufficient, which parent may serve as a more accurate informant for specific symptom domains, and how clinicians should appropriately interpret discrepant reports.

With respect to publication bias, Egger’s test indicated significant funnel plot asymmetry across the overall and gender subgroup analyses, suggesting that smaller studies with lower kappa values may be underrepresented. This pattern is consistent with a potential publication bias favoring studies reporting higher levels of parent–teacher agreement. However, given the small number of included studies (n = 11), the statistical power of Egger’s test is limited, and asymmetry may also reflect genuine heterogeneity or small-study effects. The overall conclusion of low parent–teacher agreement is robust to sensitivity analyses and is unlikely to be substantially altered by unpublished studies, as even the exclusion of any single influential study did not change the direction or statistical significance of the pooled estimate.

## 5 Limitations

Several limitations of this study must be acknowledged. First, only 11 studies met our strict inclusion criteria, resulting in relatively small and unequal sample sizes across subgroups, particularly in the clinical subgroup (n = 292 total). This limits the statistical power to detect moderate or small moderating effects and reduces the reliability of subgroup estimates. Second, while subgroup analysis by sample setting substantially reduced within-subgroup heterogeneity and identified sample setting as an important explanatory variable, substantial heterogeneity remained unexplained in the overall analysis (I² = 75.8%) and in the boys’ subgroup (I² = 63.4%). The lack of consistent reporting of potential moderators in primary studies—such as child comorbidity profiles, socioeconomic status, parental mental health, teacher-to-student ratios, and specific assessment instruments—precluded a more comprehensive quantitative exploration of heterogeneity sources. Third, the gender subgroup analyses did not include a formal statistical test of subgroup differences, and the observed numerical difference between boys (κ = 0.25) and girls (κ = 0.32) should be regarded as descriptive rather than confirmatory. Fourth, Egger’s test indicated significant funnel plot asymmetry across all analyses, suggesting that potential publication bias cannot be excluded. However, given the small number of included studies, the statistical power of these tests was limited, and the asymmetry may partly reflect genuine heterogeneity or small-study effects rather than selective publication alone. Finally, despite an updated search conducted through May 31, 2026, no additional eligible studies were identified, confirming that the available evidence base for this specific research question remains limited.

## 6 Conclusions

This study shows low agreement between parents and teachers on overall ADHD symptom ratings. Gender showed a small numerical difference in parent–teacher agreement (girls: κ = 0.32; boys: κ = 0.25), though current evidence is insufficient to support gender as a statistically robust moderator. In contrast, sample setting significantly moderated agreement levels (χ² = 13.39, df = 2, p = 0.0012), with school-based samples showing the highest concordance and clinical samples showing the lowest and non-significant agreement. Sensitivity analyses confirmed that the overall findings were robust to individual study exclusion, and potential publication bias—while suggested by Egger’s test—is unlikely to substantially alter the conclusion of low overall parent–teacher agreement given the consistency of findings across leave-one-out scenarios. These results highlight the importance of obtaining comprehensive contextual information and systematically analyzing and integrating reports from multiple informants to ensure accurate and reliable ADHD diagnoses.

## Data Availability

The original contributions presented in the study are included in the article/**Supplementary Material**. Further inquiries can be directed to the corresponding author.
